# Advances in research on gut microbiota and allergic diseases in children

**DOI:** 10.1016/j.crmicr.2025.100362

**Published:** 2025-02-18

**Authors:** Heng Ke, Hongbing Yao, Ping Wei

**Affiliations:** Department of Otolaryngology, The Children's Hospital of Chongqing Medical University, Chongqing, PR China

**Keywords:** Atopic dermatitis, Food allergy, Allergic rhinitis, Asthma, Gut microbiota, Probiotics, Short chain fatty acids

## Abstract

Epidemiological studies indicate a rising prevalence of allergic diseases, now recognized as a major global public health concern. In children, the progression of these diseases often follows the "atopic march," beginning with eczema, followed by food allergies, allergic rhinitis, and asthma. Recent research has linked gut microbiota dysbiosis to the development of allergic diseases in children. The gut microbiota, a crucial component of human health, plays a vital role in maintaining overall well-being, highlighting its potential in preventing and modifying the course of allergic diseases. This review examines the relationship between childhood allergic diseases and gut microbiota, drawing on the latest evidence. We first elaborated the concepts of allergic diseases and gut microbiota, followed by a discussion of the developmental trajectory of the gut microbiota in healthy children. This review further explored the richness, diversity, and composition of the gut microbiota, as well as specific microbial taxa associated with allergic disease. Lastly, we discussed the current status and future potential of probiotic interventions in managing pediatric allergic diseases.

## Highlights

• This study highlights the growing global burden of allergic diseases in children and their recognition as a significant public health challenge.
• It provides an overview of recent advances in understanding the interplay between gut microbiota and allergic diseases in children.
• The associations of alpha and beta diversity, specific microbial taxa, metabolites, and probiotics with pediatric allergic diseases are systematically discussed.

## Graphical abstract

**Figure:**
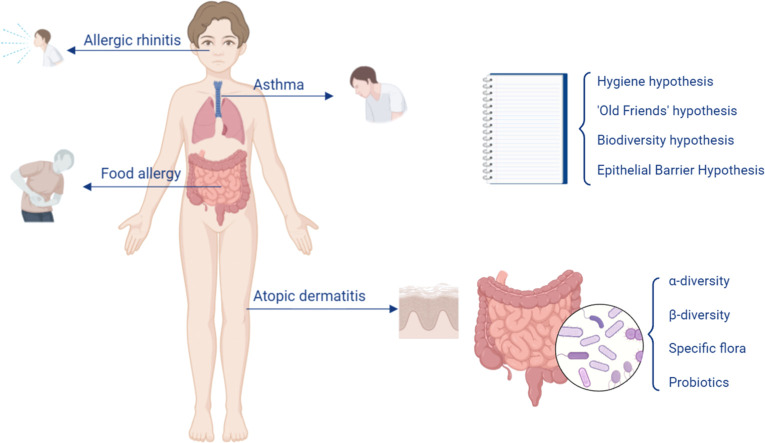

## 1 Allergic diseases

### 1.1 Basic concept of allergic diseases

**Fig. 1:**
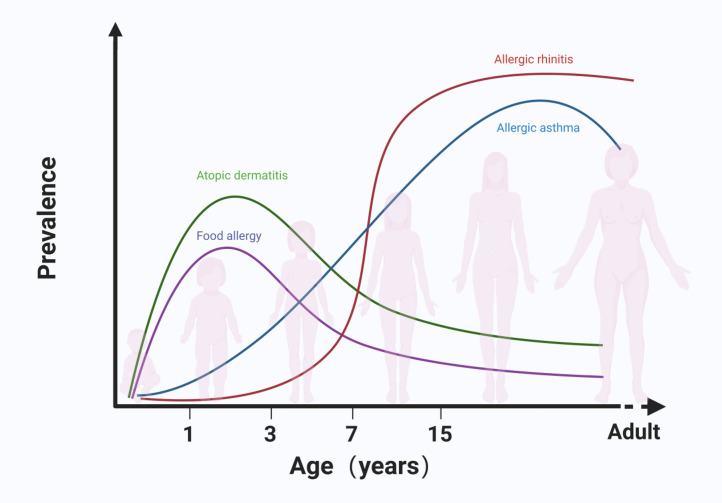
The development of allergic diseases in children follows a distinct, sequential pattern known as the Atopic March. This progression typically begins in infancy, with AD peaking in prevalence during the first two years of life. The timing of food allergy onset varies by allergen; allergies to chicken egg, and cow milk usually emerge within the first year, while allergies to wheat, soy, and peanuts tend to develop later in childhood. The incidence of AR steadily increases from early childhood through adolescence. Asthma, which exhibits significant heterogeneity reflecting diverse underlying pathophysiological mechanisms, typically first presents before the age of five. Created with BioRender.com.

Allergic diseases are characterized by an overreaction of the immune system to allergens, resulting in elevated levels of specific immunoglobulin E (IgE), which triggers clinical symptoms in various target organs of the body (Han et al., 2017). Common allergic diseases include atopic dermatitis (AD), food allergy (FA), allergic rhinitis (AR), and asthma, all of which are typically classified as type I hypersensitivity reactions mediated by IgE. These diseases often co-occur in early childhood, although their onset, remission, and progression vary in timing (Fig. 1) (Kilanowski et al., 2023). The development of allergic diseases is influenced by a complex interplay of factors, including dysregulated immune responses, chronic inflammation, tissue remodeling, and hyperreactivity of affected tissues. While current treatments can effectively manage allergic symptoms over extended periods, they do not provide a complete cure. Furthermore, the high prevalence of these conditions results in substantial healthcare costs, making allergic diseases a significant global public health challenge.

### 1.2 Epidemiology of allergic diseases

In 1819, John Bostock first reported hay fever, a form of seasonal allergic rhinitis (AR) that physicians scarcely recognized at the time (Bostock, 1819). The prevalence of hay fever began to rise after 1870 and continued to increase throughout the 20th century. Similarly, the incidence of asthma, AD, and FA has surged significantly since the 1960s, reaching epidemic levels by the 1990s (Platts-Mills, 2015) [4]. As the initial step in the atopic march, AD now ranks 15th among all non-fatal diseases and is the leading cause of disease burden among skin conditions (Ständer, 2021). A multicenter study in China found the overall prevalence of AD in infancy to be 30.48 %, with the majority of cases being mild (67.4 %) and involving facial dermatitis (72.07 %) (Guo et al., 2019). FA is another common allergic condition, affecting children at higher rates than adults. Approximately 10 % of infants under one year old may develop IgE-mediated food allergies to at least one common allergen (Osborne et al., 2011). In the United States, about 7.6 % of children suffer from FA, with peanut allergies being the most prevalent, followed by milk allergies (Gupta et al., 2018). In China, the prevalence of FA is estimated at 3.8 % among infants aged 0–1 years, confirmed through oral food challenges (OFC) (Chen et al., 2011). Questionnaire studies indicate a higher prevalence of FA in urban infants aged 0–2 years, with rates ranging from 5.5 % to 7.3 %, and egg allergy appears to be the most common, followed by cow's milk allergy (Chen et al., 2012). AR was rarely reported in the early 20th century and seemed to be more prevalent in families with higher socioeconomic status (Greiner et al., 2011). By the latter half of the 20th century, the number of primary care consultations for AR in the UK had nearly quadrupled (Fleming and Crombie, 1987). The overall prevalence of physician-diagnosed AR is 10.48 %, with a self-reported prevalence of AR at 19.93 % (Licari et al., 2023). Asthma, which emerges later in the atopic march, is closely associated with AD, FA, and AR. Currently, an estimated 334 million people worldwide suffer from asthma, with a prevalence of about 4.3 % among adults diagnosed by physicians (Papi et al., 2018). Asthma occurs slightly more frequently in children than in adults, with a higher incidence in boys than in girls (Stern et al., 2020). Furthermore, allergic diseases exhibit higher prevalence rates in industrialized nations, placing a significant economic burden on families and society (Sicherer and Sampson, 2018).

### 1.3 Environmental factors increasing the prevalence of allergic diseases

The continued rise in the prevalence of allergic diseases over the past two centuries can be attributed to their multifactorial pathogenesis, involving a complex interplay of factors such as the maternal-fetal environment, living conditions, genetic predispositions, epigenetic modifications, and immune status (Wang et al., 2023). Twin and family studies strongly support the significant role of genetics, with the concordance rate for atopic dermatitis approximately 80 % in identical twins, compared to 20 % in fraternal twins (Schultz Larsen, 1993). Genome-wide association studies (GWAS) have identified susceptibility genes related to epithelial barrier function, the immune system, and the vitamin D pathway, highlighting their role in allergic disease pathogenesis (Ober, 2021). While genetic predispositions lay the foundation, they alone cannot explain the rapid increase in allergic diseases. Environmental factors contribute by either indirectly influencing genetic predispositions or directly impacting immune system development.

Environmental changes resulting from modernization and urbanization affect susceptible genes, leading to epigenetic modifications such as DNA methylation and histone modifications, which play a role in the development of allergic diseases (Acevedo et al., 2021). These modifications suggest that genetic factors can lead to significantly different responses among individuals, even when exposed to similar environmental conditions. Furthermore, humans have coexisted with environmental microorganisms, and changes in the composition of the microbiota, which are driven by environmental factors may significantly impact immune system development, particularly innate immunity.

The rise in allergic diseases highlights the complex interplay between genetics, environmental factors, and immune system development, with several hypotheses providing insight into how modern lifestyle changes disrupt immune regulation and barrier functions. In 1989, Strachan proposed the hygiene hypothesis, which suggests that infections acquired during prenatal and childhood periods have a protective effect against the development of allergic diseases. However, improved sanitation and smaller family sizes may reduce this protective effect, contributing to the rise in allergic diseases (Strachan, 1989). Building on this, Graham Rook proposed the 'Old Friends' hypothesis in 2003, suggesting that exposure to specific commensal microbes and parasites, with which humans have co-evolved, provides essential immunoregulatory signals. These interactions help prevent immune-mediated diseases like allergies and autoimmune disorders, playing a crucial role in immune system development (Rook et al., 2004).

The 'biodiversity hypothesis' proposed by Tari Haahtela in 2013, posits that a diverse gut microbiota is essential for immune system development. Alterations in the microbiota of the gut, skin, and respiratory system—leading to a loss of symbiosis and the onset of dysbiosis—are linked to an increased risk of allergic diseases (Haahtela et al., 2013). The "Epithelial Barrier Hypothesis", introduced by Pothoven and Schleimer in 2017 for type 2 inflammatory diseases, suggests that epithelial dysfunction plays a central role in the development of allergies, autoimmune disorders, and chronic diseases (Pothoven and Schleimer, 2017). In 2021, Akdis expanded on this hypothesis, proposing that environmental changes driven by modern urban lifestyles compromise epithelial barriers in the skin, respiratory tract, and gastrointestinal mucosa. This barrier dysfunction allows microbial dysbiosis, enabling bacteria to migrate into inter-epithelial and sub-epithelial regions, potentially causing tissue micro-inflammation (Akdis, 2021). Disruption of the epithelial barrier, followed by widespread T-cell activation, is thought to play a key role in the development of atopic conditions in childhood (Han et al., 2017). These processes not only contribute to localized allergic and autoimmune diseases in affected tissues but may also have systemic effects, as the immune response to displaced bacteria can trigger the onset of other diseases (Celebi Sozener et al., 2022).

## 2 Gut microbiota

### 2.1 Concept of gut microbiota

**Fig. 2:**
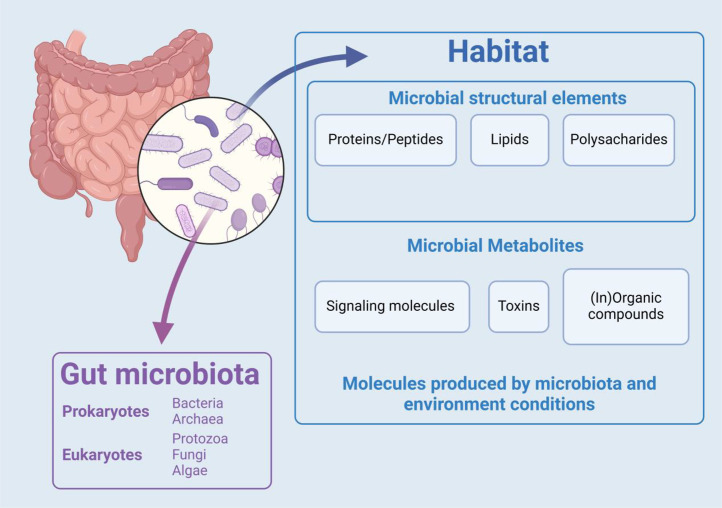
A diagram illustrating the term gut microbiome, which encompasses not only the gut microbiota but also their habitat. Created with BioRender.com.

The human body is not a sterile environment; rather, it teems with a vast and diverse community of commensal microorganisms. These microbes inhabit various body cavities and surface areas, including the digestive, respiratory, and urinary tracts, the female reproductive system, and the skin. In a healthy adult, the number of human cells is estimated at approximately 3 × 10^13^, while the number of symbiotic microorganisms is around 4 × 10^13^ (Sender et al., 2016). Among this extensive collection of symbiotic microbes, the gut microbiome stands out as the most complex and expansive ecosystem. It exemplifies the remarkable diversity of microbial life and its intimate connection to human health. The gut microbiome includes not only a variety of microbes (bacteria, archaea, fungi, and even some protozoa and algae) but also their genetic material, as well as their metabolic products, including proteins, lipids, sugars, and waste products. Additionally, the gut environment itself—comprising molecules produced by both the body and the microbes—further contributes to this intricate system (Berg et al., 2020). Together, these components form a dynamic, interactive microecosystem that exhibits variability over time and across individuals (Fig. 2). The gut microbiota contains an extensive genetic library, with approximately 22 million genes—over 700 times the size of the human genome. It plays critical roles in the body, including nutrient metabolism, regulation of immune tolerance, prevention of pathogen colonization, and modulation of the host's development and behavior (Tierney et al., 2019; Cabreiro and Gems, 2013).

### 2.2 Composition and distribution of the gut microbiota

In healthy adults, the gut microbiota is predominantly composed of bacteria, but also includes archaea, eukaryotes, and viruses. While research has identified dozens of bacterial phyla in the human gut microbiota, including Bacteroidota*,* Bacillota*,* Proteobacteria*,* Actinobacteria*,* Verrucomicrobia, and Fusobacteria, high-throughput sequencing studies have shown that the majority of bacteria in healthy adult guts belong to just a few phyla. In fact, over 99 % of the bacteria are classified into *Bacteroidota* (synonym Bacteroidota), Bacillota (synonym Firmicutes), Proteobacteria, and Actinobacteria (Eckburg et al., 2005). In healthy adults, the gut microbiome generally contains minimal to no long-term fungal residents. Fungi detected in fecal metagenomes are often derived from the mouth or food (Auchtung et al., 2018). However, under certain pathological conditions, fungi can overgrow in the gut, leading to dysbiosis. For instance, studies have observed an increased abundance of *Candida albicans* in the feces of children with inflammatory bowel disease (Chehoud et al., 2015; Sokol et al., 2017). The primary archaea in the gut microbiota is *Methanobrevibacter smithii*, and the primary viruses are bacteriophages (Lozupone et al., 2012).

The gut microbiota is influenced by a variety of factors and exists in a constant state of dynamic change. From a macroscopic perspective, there are significant differences in the healthy gut microbiota between countries, regions, diets, ethnicities, and age groups (Mahdavinia et al., 2023; Yatsunenko et al., 2012; De Filippo et al., 2010; Radjabzadeh et al., 2020). These factors need to be considered when evaluating the relationship between allergic diseases and gut microbiota. However, no significant differences in the functional richness of these microbiota have been observed. On an individual level, the abundance and diversity of the gut microbiota generally increase along the gastrointestinal tract, with site-specific variations between the mucosa and the lumen. The stomach primarily hosts the *Lactobacillaceae* family, Lactobacillaceae*,* Enterobacteriaceae, and Streptococcaceae mainly inhabit the small intestine. The colon contains the largest microbial community, densely colonized by bacteria from families such as Enterococcaceae*,* Clostridiaceae*,* Enterobacteriaceae, Bacteroidaceae, Bifidobacteriaceae, and Fusobacteriaceae (Hillman et al., 2017). This distribution is largely due to varying environmental factors in different parts of the digestive tract, including pH levels, bile acid concentration, chyme retention time, mucin characteristics, and host defense mechanisms (Donaldson et al., 2016; Barcik et al., 2020). Consequently, while fecal samples are commonly used in microbiome studies, they do not accurately represent the flora of the entire gut. The fecal microbiome most closely resembles that of the colon, but differs significantly from that of the small intestine and stomach. Additionally, its alpha diversity is lower than that of the colonic microbiome, leading to discrepancies between studies based on fecal samples and the actual composition of the gut microbiome in the host (Ahn et al., 2023).

## 3 Gut microbiota and allergic diseases in children

### 3.1 Characterization of changes in the gut microbiota with age in children

**Fig. 3:**
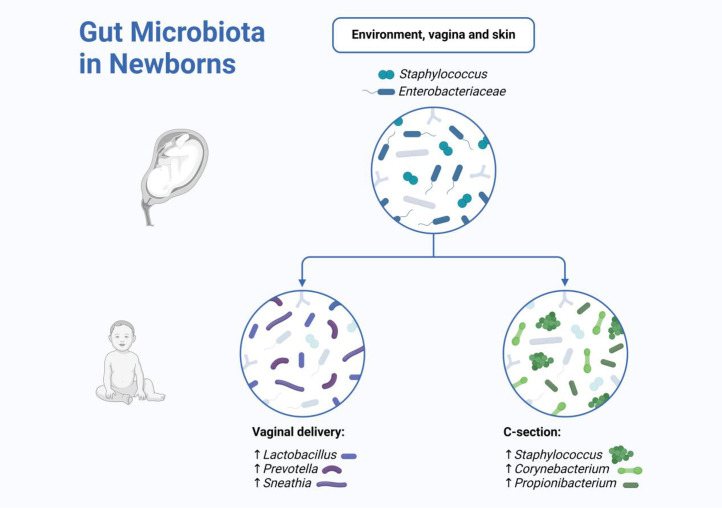
Gut microbiota in newborns. The delivery mode significantly impacts the composition of the newborn's gut microbiota. Newborns delivered vaginally have a gut microbiome rich in *Bacteroides, Bifidobacterium, Parabacteroides*, and *Escherichia/Shigella.* In contrast, cesarean-delivered newborns exhibit a gut microbiome dominated by *Staphylococcus, Corynebacterium*, and *Propionibacterium*. Created with BioRender.com.

Investigations reveal that the basic characteristics of gut microbiota changes in childhood. These changes exhibit distinct successional stages, which can be divided into three periods: a period of rapid change during infancy, a period of stabilization in adulthood, and a period of degeneration in the elderly. Among these stages, the rapidly changing gut microbiota in infancy plays a critical role in shaping immune health across the lifespan. Overall, the abundance and diversity of the gut microbiota undergo dynamic changes during early life, reflecting the growth and development of the host. Microbial colonization begins at birth, with the rupture of the amniotic sac. While microbial DNA and occasional live bacteria have been detected in the placenta, amniotic fluid, and meconium, there is no evidence of a permanent live microbial community in the uterus, placenta, fetus, or bloodstream (Aagaard et al., 2014; DiGiulio et al., 2010; Dominguez-Bello et al., 2019). The mode of delivery is a major determinant of the bacterial community composition in the newborn (Fig. 3). Newborns delivered vaginally have a gut microbiome dominated by bacteria such as *Bacteroides, Bifidobacterium, Parabacteroides*, and *Escherichia/Shigella*, which mirrors the microbial community of the mother's vaginal microbiome, predominantly consisting of *Lactobacillus, Prevotella*, or *Sneathia* (Bäckhed et al., 2015). In contrast, cesarean-delivered infants lack bacteria from the maternal vaginal community. Instead, their initial colonizers often come from the mother's skin, oral cavity, and the surrounding environment, with bacteria such as *Staphylococcus, Corynebacterium*, and *Propionibacterium* (Dominguez-Bello et al., 2010).

During the neonatal period, the gut microbiota can be categorized into one of three distinct community states, each driven by a predominant microorganism: *Bifidobaterium longum, Bifidobaterium breve*, and *Enterococcus faecalis* (Shao et al., 2024). These community states evolve in different directions. Communities dominated by *Bifidobacterium longum* or *Bifidobaterium breve* tend to follow stable assembly trajectories and confer long-term resistance to pathogenic microbial colonization. In contrast, the acquisition of a neonatal gut microbiota driven by *Enterococcus faecalis*, which is often associated with cesarean delivery and antibiotic use during pregnancy is linked to unstable assembly trajectories and a higher pathogen load. As infants age, species richness and diversity (α-diversity) of the gut microbiota increase, and substantial changes in its overall composition (β-diversity) and dominant flora are observed (Cheung et al., 2023; Lee et al., 2022). A study by M. Tanaka et al. found that gut microbiota composition changes significantly at two key stages in early life: between birth and 6 months, when Actinobacteria and *Bifidobacteria* becomes the dominant microbiota in breastfed infants; and between 6 and 12 months, a phase characterized by greater variability, with an increase in Bacillota and Bacteroidota and a decrease in Actinobacteria and Proteobacteria (Tanaka et al., 2017). This shift correlates with the transition from a *Bifidobacteria*-dominated microbiota to one that reflects solid food intake after breastfeeding. While breast milk supports the development of a healthy gut microbiota in infants, prolonged breastfeeding can delay the maturation of the infant's microbiome (Depner et al., 2020).

Studies have shown that infants with an immature gut microbiome are at greater risk of developing allergic asthma later in life (Stokholm et al., 2018). The first 1000 days of life—from conception to the second year after birth—are crucial for establishing a healthy gut microbiome. This complex community of microbes typically reaches a state closer to that of an adult microbiome around age three (Yatsunenko et al., 2012; Notarbartolo et al., 2023). Emerging evidence suggests that the gut microbiome is influenced by various factors, including antibiotic use, mode of delivery, dietary habits, exposure to infectious diseases, and breastfeeding (Bokulich et al., 2016; Mitchell et al., 2020; Wolter et al., 2021; Kim et al., 2017). Disturbances in the gut microbiome and metabolic dysregulation during early life can persist into adulthood, potentially affecting the onset and progression of allergic diseases (e.g., allergic rhinitis, asthma), autoimmune diseases (e.g., inflammatory bowel disease), and metabolic diseases (e.g., obesity) (Notarbartolo et al., 2023; Renz and Skevaki, 2021; Di Costanzo et al., 2021). From this perspective, ensuring a healthy gut microbiome is essential for managing children's health.

### 3.2 Alterations of richness, diversity and composition of the gut microbiota in children with allergic diseases

Though various studies have reported that association of several microbiota with the pediatric allergic diseases, research on the association between gut microbiota diversity and childhood allergic conditions has yielded conflicting results. AD, one of the most prevalent allergic diseases in early childhood, has been linked to reduced gut microbiota diversity in some studies, with a negative correlation between diversity levels and symptom severity (Abrahamsson et al., 2012; Nylund et al., 2015). However, most recent studies have reported no significant difference in fecal α and β diversity between children with AD under the age of 3 years and healthy controls (Cheung et al., 2023; Patumcharoenpol et al., 2023; Fan et al., 2022; Park et al., 2020). Notably, when children with AD were categorized based on symptom severity, a decrease in α diversity was observed in fecal samples from those with moderate-severe AD at 6 months of age (Lee et al., 2022). Similarly, when grouped by symptom duration, no significant difference in α diversity were found between the healthy controls and children with both the transient AD and persistent AD at 6 months of age (Park et al., 2020). Another study has also identified lower α diversity in children aged 3–12 years with AD, along with significant differences in microbial species composition compared to healthy controls (Nekrasova et al., 2024). In addition, no substantial differences in α and β diversity were found before the onset of AD, suggesting that gut microbiota composition and diversity may not predict the development of this disease (Cheung et al., 2023; Fan et al., 2022).

In the context of FA, studies have generally reported no significant differences in gut microbiota α diversity between affected children and healthy individuals (Nekrasova et al., 2024; Łoś-Rycharska et al., 2021; Savage et al., 2018; De Filippis et al., 2021). However, given the diverse allergen types and underlying mechanisms in FA, gut microbiota characteristics may vary according to the specific allergens involved. Common food allergens, such as peanuts, milk, and eggs, can trigger either IgE-mediated or non-IgE-mediated allergic responses, with some allergies persisting throughout life while others resolve with age. Furthermore, dietary restrictions imposed to avoid allergenic foods may complicate the interpretation of microbiota differences. A multicenter study of 141 children revealed higher α diversity in children with egg allergies compared to healthy controls, along with significant differences in microbial composition (Fazlollahi et al., 2018). Similarly, D. Moriki et al. revealed a significant difference in β diversity between children with milk allergies and healthy individuals. This difference narrowed once milk allergy tolerance was achieved, suggesting that the that the allergy itself may drive microbiota dysbiosis, rather than milk consumption per se (Moriki et al., 2024). Furthermore, no significant differences were observed in the gut microbiota composition between children with single-antigen and multi-antigen FA, indicating potential common features of gut dysbiosis across different FA types (De Filippis et al., 2021; Goldberg et al., 2020). Goldberg et al. further reported lower α diversity in children with IgE-mediated food allergies, along with substantial microbial compositional differences compared to healthy children (Goldberg et al., 2020). In contrast, V. M. Martin et al. analyzed fecal samples from 81 children with food protein-induced allergic proctocolitis (FPIAP), a non-IgE-mediated food allergy, and found no significant differences in α or β diversity. However, the study identified a significantly higher relative abundance of Enterobacteriaceae and a lower relative abundance of Clostridiales in the gut microbiota of children with FPIAP during the symptom persistence period (Martin et al., 2022).

For respiratory allergies (RA), such as AR and asthma, the gut microbiota is known to interact with respiratory immune system through the gut-lung axis (Budden et al., 2017). While studies in adults with AR have found reduced α diversity in gut microbiota compared to healthy individuals, similar findings in pediatric AR cases remain scarce (Liu et al., 2020; Watts et al., 2021; Zhu et al., 2020; Wan et al., 2023). In contrast, a large study found that infants with lower α diversity in their gut microbiota during the first year of life were at an increased risk of developing AR in later years (Bisgaard et al., 2011). Wan et al. observed that children with asthma exhibited higher α diversity and distinct β diversity profiles compared to healthy controls (Wan et al., 2023). Abrahamsson's research indicated that reduced α diversity in the first month of life may increase the risk of early-onset allergic asthma (Abrahamsson et al., 2014). However, no reduction in gut microbiota diversity was found in early-life fecal samples from children with asthma (Stokholm et al., 2018; Arrieta et al., 2015; Arrieta et al., 2018). Additionally, several studies reported no significant differences in α and β diversity between children with transient wheezing and healthy controls, regardless of their future asthma risk (Bannier et al., 2020; Arrieta et al., 2018; Chiu et al., 2020; Stokholm et al., 2018).

### 3.3 Association of specific flora with allergic diseases

Next, we investigated the association between specific bacterial genera and allergic diseases in children. A review of recent studies examining the gut microbiome in pediatric allergic diseases was conducted, and observed changes in gut microbiota were summarized in Table 1. Several bacterial taxa were found to differ significantly in the fecal samples of children with allergic diseases compared to healthy controls. However, the causal relationship between these altered microbial communities and the development of allergic diseases remains unclear. Furthermore, there is no consensus in the literature regarding these differences, which may be influenced by a range of factors, including study design (e.g., variations in sampling time points and sequencing methods), environmental factors (e.g., dietary and lifestyle differences, hygiene standards across regions), and host-specific factors (e.g., genetic and ethnic differences). (Manghi et al., 2024) Despite these challenges, certain genera appear frequently in studies of allergic diseases and gut microbiota, such as *Prevotella, Bacteroides*, and Ruminococcaceae, which are regarded as core species of the gut microbiota due to their significant variability in relative abundance among individuals (Costea et al., 2018).

**Table 1:** Summary tabulation of studies on the gut microbiota and allergic diseases in children from 2019 to 2024, CMA: UCG: unclassified genus.↑:increased compared to the controls ↓:decreased compared to the controls.

Year	First author	Number of patients	Country or region	Age at outcome	Age at stool sampling	Gut microbial changes	Reference
2024	Dafni Moriki	68 (32 CMA)	Greece	5–12 y	5–12 y	↓ Bifidobacterium ↓ Coprococcus catus ↓ Monoglobus ↓ Lachnospiraceae GCA-900,066,575 ↑ Oscillibacter valericigenes ↑ Negativibacillus massiliensis ↑ Ruminococcaceae incertae sedis ↑ Ruminococcaceae unclassified genus 1 ↑ Ruminococcaceae unclassified genus 2	(Moriki et al., 2024)
2024	Nekrasova, Alexandra I.	128 (49 AD, 46 FA)	Russia	3–12 y	3–12 y	AD: ↑ Pasteurellaceae ↓ Barnesiellaceae FA: ↑ Bifidobacteriaceae ↓ Barnesiellaceae ↓ Desulfovibrionaceae ↑ Ruminococcaceae ↑ Erysipelotrichaceae	(Nekrasova et al., 2024)
2023	Man Kit Cheung	112 (48 AD)	China	6, 12 mo	at birth, 1, 3, 6, 12, 18, 24, 30 mo	↑ Clostridium sensustricto 1 ↓ Bacteroides	(Cheung et al., 2023)
2023	Preecha Patumcharoenpol	62 (23 AD)	Thailand	9–30 mo	9–12mo, 18–21mo. 24–30 mo	↑ Anaerostipes ↑ Lachnoclostridium ↑ Butyricicoccus ↓ Oscillibacter ↓ UBA1819 ↓ Eisenbergiella ↑ Lactobacillus ↓ Lachnoclostridium	(Patumcharoenpol et al., 2023)
2023	Jinyi Wan	60 (23 asthma, 18 AR)	China	6–14 y	6–14 y	↑ Corynebacterium ↑ Streptococcus ↑ Dorea ↑ Actinomyces ↑ Bifidobacterium ↑ Blautia ↑ Rothia ↓ Bacteroides ↓ Alistipes ↓ Acidaminococcus	(Wan et al., 2023)
2022	Xiaoxiao Fan	36 (10 AD)	China	2 y	at birth, 6 mo, 1 y, 2 y	↑ Eubacterium xylanophilum ↑ Ruminococcus gauvreauii ↑ UCG-002 ↓ Gemella ↓ Veillonella	(Fan et al., 2022)
2022	Min-Jung Lee	346 (234 AD)	Korea	6–36 mo	6–36 mo	↓ Alistipes finegoldii ↓ unclassified Oscillibacter ↓ unclassified Alistipes	(Lee et al., 2022)
2022	Victoria M. Martin	160 (81 FA)	United States	1 y	<1 y	↑ Enterobacteriaceae ↓ Clostridiales	(Martin et al., 2022)
2021	Ewa Łoś-Rycharska	87 (59 FA and/or AD)	Poland	FA and/or AD at 0–6 mo	0–6 mo	↑ Bacteroides ↑ Parabacteroides ↓ Fusicatenibacter saccharivorans ↓ Lactococcus lactis ↓ Serratia marcescens	(Łoś-Rycharska et al., 2021)
2021	Francesca De Filippis	114 (30 RA, 55 FA)	Italy	4–7y	4–7 y	↑ Ruminococcus gnavus ↑ Faecalibacterium prausnitzii ↑ Dialister invisus ↑ Anaerostipes hadrus ↑ Blautia ↑ Parabacteroides ↓ Bifidobacterium longum ↓ Bacteroidesdorei ↓ Bacteroides vulgatus ↓ Some fiber-degrading taxa	(De Filippis et al., 2021)
2021	Khui Hung Lee	60 (33 FA)	Australia	1–7y	1–7 y	↑ Ruminococcaceae UCG-002 ↑ Eubacterium oxidoreducens group ↑ Eubacterium coprostanoligenes group ↑ Lachnospiraceae(NK4A136 and UCG-008) ↑ Bacteroides ↑ Alistipes ↑ Parabacteroides ↑ Prevotella 2 ↑ Rhodospirillaceae	(Lee et al., 2021)
2020	Michiel A G E Bannier	230 (70 asthma, 114 transient wheezing)	Dutch	Asthma at 6 y	2–4 y	↑ Gemmiger ↑ Escherichia ↓ Collinsella ↓ Dorea	(Bannier et al., 2020)
2020	S Björkander	65 (16 any allergic disease)	Sweden	Any allergic disease at 10 y	1 w, 2 w, 1 mo, 2 mo	↓ Lacticaseibacillus casei ↓ Lacticaseibacillus paracasei ↓ Lacticaseibacillus rhamnosus	(Björkander et al., 2020)
2020	Chiu, Chih-Yung	60 (20 AR, 18 asthma)	Taiwan	4–5 y	4–5 y	↓ Faecalibacterium ↓ Dorea	(Chiu et al., 2020)
2020	Yoon Mee Park	132 (22 transient AD, 26 persistent AD)	Korea	1–2 y	6 mo	Transient AD: ↓ Streptococcus ↑ Akkermansia Persistent AD: ↓ Clostridium ↓ Akkermansia ↑ Streptococcus	(Park et al., 2020)
2020	Michael R. Goldberg	233 (175 FA)	Israel	4–18 y	4–18 y	↑ Collinsella aerofa ↑ Dorea formicigenerans ↑ unclassified Methanobrevibacter ↑ Blautia obeum ↑ Coprococcus catus ↓ Prevotella copri ↓ Bifidobacterium adolescentis	(Goldberg et al., 2020)
2019	K Simonyté Sjödin	93 (21 allergic disease at 8 y)	Sweden	Any allergic disease at 8 y	4, 6, 13 mo; 8 y	↓ Bacteroides ↓ Coprococcus ↓ Enterococcus ↓ Lachnospira ↓ Lactobacillus ↓ Prevotella ↓ Ruminococcus ↑ Bifidobacterium	(Simonyté Sjödin et al., 2019)
2019	Chih-Yung Chiu	85(27 rhinitis, 34 asthma)	Taiwan	AR or asthma at 4–7 y	4–7 y	↓ Faecalibacterium ↓ Roseburia ↓ Dorea ↓ Dialister ↓ SMB53	(Chiu et al., 2019)

Cow's milk allergy, RA: Respiratory allergy, AD: Atopic dermatitis, FA: Food allergy, AR: Allergic rhinitis.

*Bifidobacterium* is a noteworthy genus in the context of allergic disease development in children. For instance, *B. bifidum* has been shown to induce the production of Foxp3+ Treg cells, which exhibit potent anti-inflammatory effects (Verma et al., 2018). It has been suggested that neonatal gut colonization by *Bifidobacterium* as the predominant species promotes a more stable microbiota and stronger resistance to pathogen colonization. In contrast, children with a non-*Bifidobacterium*-dominant gut microbiota early in life may have an increased risk of developing asthma later stages of life (Shao et al., 2024). However, several studies have reported varying levels of *Bifidobacterium* in the fecal samples of allergic children compared to healthy controls, which could be attributed to the common use of yogurt and probiotics. While *Bifidobacterium* ingestion is frequently associated with these probiotics, it is difficult to establish successful gut colonization due to the resistance posed by the resident microbiota (Laursen et al., 2017). Additionally, not all *Bifidobaterium*. spp play a crucial role in childhood allergic diseases. Although *B. longum* and *B. breve* dominate the neonatal gut microbiota, the relative abundance of *B. longum* subspecies, such as *B. infantis*, is typically low (Lee et al., 2022; Ranjan et al., 2016). Since multiple distinct species exist within the *Bifidobacterium* genus, the colonization and function of these closely related species can vary substantially. Therefore, more precise sequencing methods (e.g., whole-genome sequencing, WGS) are recommended to distinguish between species (Stokholm et al., 2018).

In children with dust mite-induced allergic rhinitis, the relative abundance of *Dorea* and *Faecalibacterium* was negatively correlated with fecal IgE levels and strongly correlated with *Leptotrichia spp*. in the airways (Chiu et al., 2020). A reduction in *Faecalibacterium spp*., accompanied by an increase in *Escherichia* spp. in the gut, was significantly associated with a higher risk of asthma. It is important to note that gut microbes play different roles at different stages of life. For example, while *Bacteroides, Prevotella*, and *Coprococcus* have been associated with allergic disease from 6 months to 8 years of age, other taxa, such as *Ruminococcus*, lose their beneficial effects during the first year of life (Simonyté Sjödin et al., 2019). A study found significantly higher relative abundances of *Gemmiger* and *Escherichia* in wheezing children who developed asthma by the age of 6 years. Specifically, *Escherichia coli* was found to be 4.6 times more abundant in these children compared to healthy controls, highlighting its potential role in asthma development (Bannier et al., 2020).

A prospective cohort study by S. Björkander et al. found a low relative abundance of *Lactobacillus* in the fecal samples of children who later developed allergic diseases, suggesting that *Lactobacillus* may play a protective role in the development of such conditions (Björkander et al., 2020). In an eight-year cohort study by K. Simonyte Sjodin et al., involving 93 children, 16S rRNA gene sequencing of fecal samples collected at 4, 6, and 13 months, as well as at 8 years of age, revealed consistent findings. Compared to healthy controls, allergic children exhibited persistently lower relative abundance of *Bacteroides, Prevotella*, and *Coprococcus* in their gut microbiota. However, *Ruminococcus* was only transiently reduced at 6 months of age, with no significant differences observed at later time points (Simonyté Sjödin et al., 2019). Additionally, the relative abundance of Lachnospiraceae in fecal samples from 2 to 12 month-old children was significantly higher in those who later developed allergic diseases, compared to non-allergic infants. Further studies have suggested that the elevated relative abundance of *Ruminococcus gnavus* within the Lachnospiraceae family is closely associated with the onset of allergic diseases (Chua et al., 2018). Multi-omics analyses also showed a significant decrease in the relative abundance of *R. gnavus* and a reduction in intestinal acetate in the early-onset persistent phenotype, which may be linked to ACSS2, JAK-STAT signaling, and systemic T helper-cells 2 (Th2) inflammation (Bao et al., 2021).

### 3.4 Short-chain fatty acids and allergic diseases in children

The intestinal flora influences the development of allergic diseases through various metabolites, with short-chain fatty acids (SCFAs) being key factors. SCFAs are fatty acids with six or fewer carbon atoms, including formate, acetate, propionate, butyrate, valerate, caprate, isovalerate, isobutyrate, and isocaprate. Among these, acetate, propionate, and butyrate are the most abundant in the human intestine, usually accounting for >90 % of the total SCFAs, while the other fatty acids account for a smaller proportion (Fusco et al., 2023). SCFAs can be obtained either directly from food or produced by fermentation of dietary fiber by intestinal bacteria. However, most of the SCFAs are still mainly produced through the fermentation process of intestinal flora. The ratio of acetate, propionate, and butyrate in the human gut is approximately 3:1:1. The substrates, synthetic enzymes, and synthetic pathways required for their synthesis have been detailed in previous studies (Hays et al., 2024; Ríos-Covián et al., 2016). Acetate production occurs primarily through the fermentation process of sugars (e.g., glucose) to produce acetic acid and lactic acid (or ethanol) with the release of energy (ATP). Common bacterial groups involved in this process include *Bifidobacterium* and *Bacteroides*. Propionate is mainly produced via the succinate pathway, mainly by several bacteria of the Bacteroidota and Bacillota, e.g. *Prevotella*. Synthesis of butyrate relies on the butyryl-CoA: acetyl-CoA transferase pathway, involving bacteria such as *Faecalibacterium, Eubacterium*, and *Roseburia*. Propionate mainly provides energy to colonic epithelial cells and support gluconeogenesis to the liver, while acetic acid and butyric acid are mainly involved in lipid biosynthesis (den Besten et al., 2013).

Emerging evidence highlights the critical role of SCFAs in modulating immune responses and maintaining gut homeostasis, particularly in pediatric allergic diseases. In-vivo studies have demonstrated that propionate and butyrate (but not acetate) inhibit histone deacetylase (HDAC) activity, leading to increased histone acetylation levels. This epigenetic modification opens up the chromatin structure and promotes the transcription of the Foxp3 gene (a key transcription factor for Treg cells), which promotes the differentiation of Treg cells (Furusawa et al., 2013; Smith et al., 2013). Treg cells are essential for maintaining immune tolerance and suppressing excessive Th2 responses, key mechanisms in allergic diseases. Meanwhile, a high-fiber diet and acetate intake enahnce the acetylation of Foxp3 (but not by inhibiting HDAC activity), which in turn promoted the formation of highly suppressive Treg cells, while decreasing IL-4, IL-5, and IL-13 secretion, and inhibiting IgE-mediated RA (Thorburn et al., 2015). The protective effect of SCFAs against allergic diseases has been partially confirmed in clinical studies. Reduced levels of SCFAs have been found in the feces of children with FA or RA compared to controls (De Filippis et al., 2021; Goldberg et al., 2020). In addition, serum SCFAs levels were low in pediatric and adult patients with FA (Goldberg et al., 2020; Tian et al., 2022). Moreover, serum propionate levels were negatively correlated with serum Th2 cytokines, and serum-specific IgE concentrations (Tian et al., 2022). In another prospective study, infants with higher levels of fecal butyrate and propionate at 1 year of age had a lower risk of allergic disease and sensitization later in life (Roduit et al., 2019). A systematic review by Mari Sasaki et al. also indicated that the three main SCFAs (acetate, propionate and butyrate) in the first years of life have a significant protective effect on the atopic dermatitis in childhood (Sasaki et al., 2024). Despite promising findings, an in-depth meta-analysis is lacking for the time being to quantitatively analyze all the existing studies due to methodological variability across studies.

### 3.5 Probiotics and allergic diseases

Finally, Given the close association between allergic diseases and dysbiosis in children, the possibility that oral probiotics could correct the dysfunctional gut microbiota and alleviate or even treat allergic diseases has been a topic of interest. Several animal studies have demonstrated that specific bacterial preparations can improve allergy symptoms. For instance, Wenwei Lu et al. showed that in mice with AD, treatment with *Bifidobacterium longum* CCFM1029 alleviated symptoms by activating immune signaling pathways mediated by tryptophan-derived metabolites such as indole-3-aldehyde (Fang et al., 2022). Another study found that oral administration of *Lactobacillus rhamnosus* or *Bifidobacterium lactis* (Bb-12) to neonatal mice inhibited subsequent allergen sensitization and airway inflammation. A small randomized controlled trial (RCT) involving 31 adult patients revealed that oral administration of *Bifidobacterium lactis* Probio-M8 for three months, in conjunction with asthma medication, significantly reduced alveolar nitric oxide levels and markedly improved asthma symptoms (Liu et al., 2021).

A number of clinical studies have also suggested that oral administration of probiotic preparations may help alleviate symptoms in children with allergic diseases. For example, infants with cow's milk allergy who were fed an extensively hydrolyzed casein formula enriched with *Lactobacillus rhamnosus* GG exhibited improved tolerance to cow's milk compared to those who were only fed the extensively hydrolyzed formula (Berni Canani et al., 2012). Moreover, the formula supplemented with *Lactobacillus rhamnosus* GG increased the relative abundance of butyrate-producing bacteria in the gut, leading to higher fecal butyrate levels, which have protective effects against allergic diseases (Berni Canani et al., 2016). In another study, oral administration of *Lactobacillus rhamnosus* IDCC 3201 tyndallizate (RHT3201) to children with moderate AD was shown to effectively relieve skin irritation (Jeong et al., 2020). Despite these promising findings, there remains a lack of conclusive evidence that probiotics can prevent the development of allergic diseases. For example, it has been suggested that oral administration of *Lacticaseibacillus rhamnosus* HN001 and various *Bifidobacterium* species to pregnant women does not reduce the risk of allergic diseases in their offspring (Komulainen et al., 2023). Similarly, continuous oral administration of probiotics for two years to preterm infants did not reduce the risk of developing allergic diseases (Plummer et al., 2020).

A separate meta-analysis on the treatment of AD in children has shown that single-strain Lactobacillus probiotics, particularly *Limosilactobacillus fermentum*, are more effective in treating AD compared to other strains such as *Lactiplantibacillus plantarum, Lacticaseibacillus paracasei*, or *Lacticaseibacillus rhamnosus*. Key factors for successful AD treatment include selecting probiotic strains with demonstrated efficacy, extending the duration of treatment, and initiating probiotic therapy early in the disease course (Fijan et al., 2023). In a meta-analysis by Cemal Cingi, which included 22 RCTs with 2242 patients aged 2–65 years suffering from seasonal or perennial AR, regular oral administration of various probiotics (including *lactobacilli, bifidobacteria*, and their mixtures) for 4 weeks to 12 months significantly alleviated AR symptoms compared to a placebo. Specifically, the *Lactobacillus paracasei* LP-33 strain significantly improved symptoms in seasonal AR patients. Other strains, such as KW3110T and HF.A00232, were found to be more effective than *Lactobacillus rhamnosus* in alleviating AR symptoms. While *Lactobacillus rhamnosus* shows promise in treating AD and FA, these newer strains have been associated with reduced nasal symptoms and lower eosinophil levels in the blood in individuals with AR (Güvenç et al., 2016). Additionally, a meta-analysis involving 28 studies highlighted that oral probiotic supplementation may be helpful in improving AR symptoms and enhancing quality of life, despite variations in subgroup analyses. This improvement may be linked to a shift in the immune system's response, reflected by an increase in the Th1/Th2 ratio. However, no significant difference in IgE levels was observed between the probiotic and placebo groups (Luo et al., 2022).

## 4 Discussion

This review provides an overview of the latest research on childhood allergic diseases and their relationship with the gut microbiome. Despite notable progress, significant contradictions persist within the current body of literature. These include inconsistencies in the reported changes in the relative abundance of specific gut microbiota, as well as differences in α- and β-diversity between children with allergic diseases and healthy individuals. Nonetheless, the roles of certain key bacterial taxa are gradually being elucidated. However, conventional sequencing methods, which typically provide genus-level resolution, are limited in their ability to capture the full complexity of microbial communities. Given that species within the same genus can exhibit substantial functional and abundance differences, further research at the species level is warranted. To achieve this, high-resolution sequencing techniques, such as metagenomic sequencing, should be employed wherever feasible (Wu et al., 2024). Moreover, metabolites derived from the gut microbiome, particularly SCFAs like butyrate, play a crucial role in the development of allergic diseases. Regulation of butyrate levels has been shown to influence the inflammatory response in model systems. Studies targeting SCFAs metabolism may hold promise in preventing disease onset and maintaining remission in immune-mediated diseases (Mann et al., 2024).

Despite promising research suggesting that probiotics could alleviate allergy symptoms, the overall effectiveness of this approach remains inconsistent. Variability in research quality, including differences in inclusion/exclusion criteria, sample sizes, probiotic strains, dosages, and supplementation duration, leads to significant heterogeneity in findings (D'Elios et al., 2020).Therefore, further investigation, particularly large-scale clinical trials, is necessary to identify the optimal timing and conditions for probiotic supplementation in preventing childhood allergies. The current understanding of the gut microbiome's influence on health and disease has evolved from focusing on individual bacterial strains to examining the entire microbial community and its complex interactions. While next-generation gene sequencing technology remains the most common approach for studying gut microbiota, it typically analyzes fecal samples, which may not fully represent the microbial communities present throughout the entire gastrointestinal tract. Differences between fecal samples and the actual gut microbiota may lead to potential misinterpretations regarding the gut microbiome's association with various diseases.

## Author contributions

All authors declare that they have made substantial contributions to the conception and design, literature review and interpretation of the manuscript and given final approval of the version to be published.

## Ethics statement

Ethics approval does not apply to this work given that this is a literature review, and no patient information is disclosed.

## Authors’ consent

All authors give their consent to the publication of this manuscript.

## CRediT authorship contribution statement

**Heng Ke:** Data curation, Writing – original draft, Visualization. **Hongbing Yao:** Conceptualization, Supervision, Writing – review & editing. **Ping Wei:** Conceptualization, Supervision, Writing – review & editing.

## Declaration of competing interest

The authors declare that they have no known competing financial interests or personal relationships that could have appeared to influence the work reported in this paper.

## Data Availability

No data was used for the research described in the article.
